# The K153R Polymorphism in the Myostatin Gene and Muscle Power Phenotypes in Young, Non-Athletic Men

**DOI:** 10.1371/journal.pone.0016323

**Published:** 2011-01-20

**Authors:** Catalina Santiago, Jonatan R. Ruiz, Gabriel Rodríguez-Romo, Carmen Fiuza-Luces, Thomas Yvert, Marta Gonzalez-Freire, Félix Gómez-Gallego, María Morán, Alejandro Lucia

**Affiliations:** 1 Department of Biomedicine, Universidad Europea de Madrid, Madrid, Spain; 2 Unit for Preventive Nutrition, Department of Biosciences and Nutrition at NOVUM, Karolinska Institutet, Stockholm, Sweden; 3 INEF, Universidad Politécnica de Madrid, Madrid, Spain; 4 Hospital Universitario 12 de Octubre, Madrid, Spain; University of Las Palmas de Gran Canaria, Spain

## Abstract

The Lys(K)153Arg(R) polymorphism in exon 2 (rs1805086, 2379 A>G replacement) of the myostatin (*MSTN*) gene is a candidate to influence skeletal muscle phenotypes. We examined the association between the *MSTN* K153R polymorphism and ‘explosive’ leg power, assessed during sprint (30 m) and stationary jumping tests [squat (SJ) and counter-movement jumps (CMJ)] in non-athletic young adults (University students) [n = 281 (214 men); age: 21–32 years]. We also genotyped the *MSTN* exonic variants E164K (rs35781413), I225T, and P198A, yet no subject carried any of these variant *MSTN* alleles. As for the K153R polymorphism, we found only one woman with the KR genotype; thus, we presented the results only for men. The results of a one-way ANCOVA (with age, weight and height entered as covariates) showed that men with the KR genotype (n = 15) had a worse performance in vertical jumps compared with those with the KK genotype [SJ: vertical displacement of center of gravity (CG) of 35.17±1.42 vs. 39.06±0.39 cm, respectively, P = 0.009; CMJ: vertical displacement of CG of 36.44±1.50 vs. 40.63±0.41 cm, respectively, P = 0.008]. The results persisted after adjusting for multiple comparisons according to Bonferroni. Performance in 30 m sprint tests did however not differ by K153R genotypes. In summary, the *MSTN* K153R polymorphism is associated with the ability to produce ‘peak’ power during muscle contractions, as assessed with vertical jump tests, in young non-athletic men. Although more research is still needed, this genetic variation is among the numerous candidates to explain, alone or in combination with other polymorphisms, individual variations in muscle phenotypes.

## Introduction

Some gene polymorphisms are candidates to explain individual variations in muscle phenotypes. The myostatin (*MSTN* or growth differentiation factor 8, *GDF8* [MIM601788]) gene [1] is receiving growing attention in the last years. The *MSTN* gene encodes myostatin, a skeletal muscle-specific secreted peptide that functions mainly to modulate myoblast proliferation and thus muscle mass and strength [2]. Variants of the *MSTN* gene are associated with muscle hypertrophy phenotypes in a range of mammalian species, most notably cattle [3], [4], dogs [5] and mice [2]. A *MSTN* polymorphism was recently associated with sprinting ability and racing stamina in thoroughbred horses [6]. The myostatin-null mouse model also provides insights into the physiological role of this protein. Besides its function in reducing sarcopenia [7], it appears that myostatin also regulates the structure and function of tendon tissues, as the stiffness of tendons is 14 times higher in myostatin-deficient mice than in their wild type controls [8].

Variations in the *MSTN* gene, as well as myostatin inhibition, can also have functional consequences in humans (see below). The potential association between *MSTN* variations and muscle mass phenotypes is best exemplified in the study by Schuelke *et al*. [9]. They reported the case of 4-year old child with both copies of the *MSTN* gene carrying a mutation (g.IVS1+5g→a transition in the splice donor site in intron 1) that results in a premature stop codon and failure to synthesize a mature, functioning protein. The child exhibited extraordinary muscle development for his age and precocious physical prowess. Systemic treatment with the myostatin inhibitor MYO-029 provides an adequate safety margin and can induce improvements in the muscle strength/function of adult patients with muscular distrophies [10]. As this type of treatment would be likely to also stimulate muscle growth in healthy humans, myostatin manipulation could be among the next generation of doping in elite sports [11].

Of the identified *MSTN* variations in humans, the Lys(K)153Arg(R) polymorphism located in exon 2 (rs1805086, 2379 A>G replacement) is one candidate to influence skeletal muscle phenotypes [12]. The Lys(K)153Arg(R) aminoacid replacement is found within the active mature peptide of the myostatin protein; it could theoretically influence proteolytic processing with its propeptide, or affinity to bind with the extracellular activin type II receptor (ActRIIB). The latter results in intracellular activation the SMAD pathway, through which myostatin induces myoblast proliferation [13] and differentiation [14], and thus muscle mass [15]. The frequency of the mutant R allele is of about 3–4% among Caucasians, with a frequency of mutant homozygotes (RR) below 1% [12], [15], [16]. Such low allelic frequency certainly limits the possibility of studying large groups of people carrying the R variant. To date, published data on the *MSTN* K153R polymorphism and human muscle phenotypes have yield controversial results, partly attributable to inter-ethnic and gender differences. Kostek et al. [15] recently found an association between the variant *MSTN* 153R allele and maximal isometric contraction of the elbow muscle flexors in African-American young adults of both genders, yet not in Caucasians. Previous studies reported no significant effect of *MSTN* variants on the muscle mass response to strength training of either Caucasians or African Americans of both genders, including World-class body-builders and elite power lifters [12]. Although, in another study *MSTN* genotypes did not explain differences in the hypertrophic response to strength training in adults of both genders, when women were analysed separately, the 153R allele was associated with a greater muscle hypertrophic response to training [17]. The *MSTN* K153R polymorphism can also affect muscle phenotypes in the elderly [16], [18], [19]. For instance, Seibert et al. reported lower muscle strength in old African American women (n = 54, 70–79 years) who carried the variant 153R allele [18].

No study has yet assessed the association between *MSTN* genotypes and muscle power during naturally occurring movements, e.g. jumping and sprinting tasks. It was the purpose of our study to examine the association between the *MSTN* K153R polymorphism and ‘explosive’ leg power of non-athletic young adults, as assessed during specific jumping and sprint tests. We hypothesized that the 153R allele is associated with decreased performance in the aforementioned tests. We also genotyped the *MSTN* exonic variants E164K (rs35781413), I225T and P198A because they also seem to cause amino acid replacements in the gene product (myostatin) expressed in human skeletal muscle [20].

## Methods

### Ethics statement

The Medical Ethics Committee of *Universidad Europea de Madrid* (Madrid, Spain) approved the study design, study protocols and informed consent procedure. All participants provided written informed consent.

### Subjects

The study sample comprised 281 healthy young adults (University students) [mean(SD) age: 21(2) years (range: 21, 32)] of both genders (214 men, 67 women) who took part in a previous study [21]. Inclusion criteria were to be free of any diagnosed cardiorespiratory disease, and not to be engaged in competitive sports such as (i) formal, supervised ‘power’ (e.g. weight lifting or alpine skiing) or jumping oriented type of training (e.g. plyometrics, volleyball or basketball) or (ii) endurance training (e.g. running, swimming of bicycling), that is, performing less than one (power) or three (endurance) structured weekly training sessions within the last year. All participants were of the same Spanish (Caucasian) ancestry for at least 3 generations.

### Genotype assessment

Sequences corresponding to the E164K, I225T, K153R and P198A variants were amplified during Spring 2009 by the polymerase chain reaction (PCR) in the Genetics Laboratory of the *Universidad Europea de Madrid*. The primers used were 5′-GAAAACCCAAATGTTGCTTC-3′ and 5′-TGTCTAGCTTATGAGCTTAGGG-3′. The PCR conditions were as follows: initial denaturing at 95°C 10 min; 35 cycles at 95°C 1 min, 52°C 45 s, 72°C 1 min and a final extension at 72°C 5 min.

The resulting PCR products were genotyped by single base extension (SBE) [22]. The primers used for E164K, I225T K153R, and P198A were 5′-CAAACACTGTTGTAGGAGTCT-3′, 5′-CTGAATCCAACTTAGGCA-3′, 5′-TTTAATACAATACAATAAAGTAGTAA-3′, and 5′-TTTTTTTTATCTCTGAAACTTGACATGAAC-3′ respectively. The PCR SBE conditions were: 96°C 10 s; 25 cycles at 50°C 5 s and 60°C 30 s. The resulting PCR products were detected in an ABI PRISM (Applied Biosystems, Foster City, CA).

### Phenotype assessment

Assessment of leg muscle ‘explosive’ power was performed during spring 2008 in the same location (UEM) and all the tests were supervised by the same researchers, as detailed elsewhere [21]. Squat (SJ) and counter-movement jump (CMJ) tests were performed using an infrared contact timing platform (Globus Ergo Tester, Codognè, Italy) to evaluate leg muscles' ability to produce ‘explosive’ power [23]. Both tests were performed three times (each separated by a two-minute rest period) and the best score was retained.

Subjects also performed a 30 m sprint test in an indoor rubberized track under two conditions: (1) starting from the stationary (standing) position [23] and (ii) starting with a previous 15 m run (running) thereby allowing achieving higher speeds in the first meters of the test [24]. The difference in performance time between both tests (at 15 m and 30 m respectively) was used as an index of subject's ability to produce acceleration, i.e. lesser difference implies higher acceleration capacity. We used photoelectric gates at 0, 15 and 30 meters to start and stop a digital timer. We previously showed the reliability of the aforementioned tests for explosive leg muscle power assessment in a subgroup of the present subjects [21].

### Statistical analysis

We tested Hardy-Weinberg equilibrium using a χ^2^ test. We analysed the differences in the study phenotypes among genotypes (KK vs. KR) of the K153R (rs1805086) polymorphism by one-way analysis of covariance, where the polymorphism was entered as a fixed factor, the phenotype was entered as a dependent variable, and age, weight and height were entered as covariates. We calculated the effect size statistics as Cohen's *d* (*standardized mean differences*) and 95% confidence interval [25]. Values of *d* 0.2, 0.5 and 0.8 are considered small, medium and large effects, respectively. We used Bonferroni & Holm method to correct for multiple testing [26]. All statistical analyses were performed using the PASW (v. 18.0 for WINDOWS, Chicago).

## Results

We detected no failures in sample collection and DNA acquisition. Genotyping success rate was >99.29% (two missing data, one man and one woman).

Genotype distributions met Hardy-Weinberg equilibrium (P = 0.59). No subject carried the variant alleles E164K, I225T, or P198A. We found only one woman with the KR genotype; thus, we present the results only for men. Table 1 shows the association between the K153R polymorphism and study phenotypes in men. We observed that men with the KR genotype had a worse performance in vertical jump (SJ and CMJ) compared with those with the KK genotype. The results persisted after adjusting for multiple comparisons. The variance explained ranged from 5 to 10%. Effect size statistics, as measured by the Cohen's *d*, indicated a medium effect size. Performance in sprint tests did not differ by K153R genotypes.

**Table 1:** Mean estimates of study phenotypes by genotypes of the K153R (rs1805086) polymorphism in the *GDF8* gene in men.

	KK (n = 201)		KR (n = 15)		P	R^2^	Cohen's *d*	(95%CI)
**Vertical Jump Tests**								
***SJ***								
Flight time (s)	563.09	(38.95)	533.94	(43.13)	0.007	0.100	0.74	0.461–1.014
Vertical displacement of CG (cm)	39.06	(5.44)	35.17	(5.49)	0.009	0.095	0.71	0.431–0.983
***CMJ***								
Flight time (s)	574.35	(39.62)	543.29	(46.20)	0.005	0.057	0.76	0.483–1.038
Vertical displacement of CG (cm)	40.63	(5.67)	36.44	(5.92)	0.008	0.053	0.72	0.446–0.999
**Sprint Tests**								
***30m running start***								
Time at 15 m (s)	1.92	(0.14)	1.91	(0.08)	0.684	0.049	0.11	−0.159–0.378
Time at 30 m (s)	3.76	(0.21)	3.75	(0.17)	0.987	0.065	0.00	−0.263–0.273
***30m standing start***								
Time at 15 m (s)	2.54	(0.12)	2.56	(0.13)	0.533	0.002	−0.17	−0.436–0.101
Time at 30 m (s)	4.41	(0.19)	4.44	(0.23)	0.559	0.014	0.16	−0.111–0.425

Values are means (standard deviation).

P values related to group differences (one way analysis of covariance after adjusting for age, weight and height).

Cohen's *d* (standardized mean differences) and 95% confidence interval (CI). Values of d 0.2, 0.5 and 0.8 are considered small, medium and large effects, respectively.

Abbreviations: SJ, squat jump; CMJ, counter-movement jump; CG, centre of gravity.

## Discussion

The main, novel finding of our study was that the variant 153R allele of the *MSTN* K153R polymorphism is associated with decreased jumping performance in young non-athletic men. Sprinting (running) ability was however unaffected by the *MSTN* K153R genotypes. Although more research is needed, and while keeping in mind that exercise-related phenotypes are likely polygenic, our data give support for a role of the *MSTN* K153R polymorphism in explaining, at least partly, individual variations in the humans' capacity for muscle ‘peak’ power generation. In contrast, in the present cohort of subjects we previously found no association between performance in the jump/sprint tests and the R577X polymorphism in the gene (*ACTN3*) encoding α-actinin-3 [21]. This variation is thought to play an important role in the muscles' ability to produce high power, at least in elite athletes [27].

We assessed ‘explosive’ muscle power by means of jumping and sprinting tests, which are naturally occurring multi-joint movements in humans that involve the coordinated participation of the majority of lower limb muscles [28], [29]. We believe this is in fact a strength of our study versus previous research in the field of genetics and exercise-related phenotypes that used other tests for muscle power assessment, for instance, maximal concentric muscle work during single-joint movements (e.g. flexor elbow contractions) at relatively low angular velocities (≤120°·s^−1^) [30]. However, during actual natural high muscle power actions such as the sprint and jumps performed by our subjects, angular velocities at the hip or knee joints can approach 800–1000°·s^−1^ [31]. To note is that our findings are partly limited by the fact that we did not assess muscle mass in our cohort, and therefore we could not determine whether the influence of *MSTN* K153R genotypes on muscle power is mediated by its expected effects on muscle mass. Finally, the finding that the *MSTN* K153R polymorphism was associated with vertical jump performance but not with sprint performance warrants further investigation. Although both tests are thought to determine muscle power performance, stationary jumps and running sprints are determined by different factors. The critical factor during running sprints, owing to the short duration of the foot contact on the ground, is the rate of force development, which in turn is determined by many factors such as muscle fibre type, synchronization of motor units, tendon stiffness, or lean mass of lower extremities [32]. In contrast, the ability of leg muscles (quadriceps) to produce power during the concentric phase of muscle contraction is the main factor affecting stationary vertical jumps as the ones we used here [33]. The elastic properties of tendons can also influence jump ability, at least in the case of CMJ. Compared with a stiffer muscle tendon complex (MTC), people with a more compliant MTC should be more efficient in utilizing elastic strain energy during jumps [34], [35]. The fact that our KR subjects showed worst jumping performance than their wild-type KK counterparts could be associated, at least partly, with a potential role of myostatin in tendon structure. Myostatin-deficient mice showed indeed 14 times higher tendon stiffness than wild-type mice [8]. Further research is needed to determine the possible association between *MSTN* polymorphisms and tendon characteristics in humans. Up to date, published data on the *MSTN* K153R polymorphism and human muscle phenotypes (at baseline or in response to training) have yield controversial results, at least in adults of young or medium age. Inter-ethnic differences in allele frequencies, gender-related differences and the low allelic frequency of the 153R allele (limiting the possibility of studying large groups of people carrying the R variant) are important reasons for controversy. Kostek et al. [15] recently found an association between the *MSTN* 153R allele and maximal isometric contraction of the elbow muscle flexors in a group of 23 African-American young adults of both genders, yet this association was not corroborated in a much larger cohort of Caucasian young adults (n = 509, also men and women). Maximal dynamic contraction (one repetition maximum) was also unaffected by *MSTN* genotypes in both cohorts. Ferrell et al. [12] reported no significant effect of the *MSTN* variants we studied here on the muscle mass response to strength training in either Caucasians or African Americans (n = 153 men and women). In another study [17], *MSTN* genotypes did not explain differences in the hypertrophic response to strength training in 32 adults (age range: 21–75 years) of both genders studied as a group; yet, when women were analyzed separately the 153R allele was associated with a 68% larger increase in muscle volume in response to training. Thomis et al. [30] reported similar values in elbow flexor strength at baseline or in response to training in a young adult with the 153KR genotype compared with those with the 153KK genotype. Evidence for the putative influence of the *MSTN* K153R polymorphism on muscle phenotypes is probably stronger in the elderly [16], [18], [19]. Notably, in a cohort of old African American women (n = 54, 70–79 years). Seibert et al. [18] reported lower muscle strength (hip and knee flexion and handgrip strength combined) in those who carried the 153R allele. We recently reported lower muscle mass/function in a very old woman (age 96 years) with the very rare *MSTN* 153RR genotype compared to her age-matched referents with the 153KK genotype [19].

Although more research is needed, the putative effect of the K153R polymorphism on muscle phenotypes is due to its potential to alter the function of the *MSTN* gene [12]. Myostatin enters the bloodstream as a latent precursor protein; it then undergoes a proteolytic process to become a mature peptide (free from the propeptide) that binds to extracellular activin type II receptor (ActRIIB) [15]. Binding of myostatin to ActRIIB induces intracellular activation of SMAD proteins and, through the SMAD pathway, myostatin modulates myoblast proliferation [13] and differentiation [14], and thus ultimately muscle mass [15]. The Lys(K)153Arg(R) aminoacid replacement is found within the active mature peptide of the myostatin protein, and could theoretically influence (i) proteolytic processing with its propeptide or (ii) affinity to bind with ActRIIB [36], [37]. This in turn would result in inability of myostatin to modulate muscle mass/power [15].

In summary, the *MSTN* K153R polymorphism is associated with the ability to produce ‘peak’ power during muscle contractions, as assessed with vertical jump tests, in young non-athletic men. Thus, although more research is still needed, this polymorphism is among the numerous candidates to explain, alone or in combination with other polymorphisms, individual variations in muscle phenotypes.
